# Balancing public health and privacy rights: a mixed-methods study on disclosure obligations of people living with HIV to their partners in China

**DOI:** 10.1186/s12954-023-00920-9

**Published:** 2024-02-04

**Authors:** Ziyi Xie, Zhizhuang Duan

**Affiliations:** 1 Macao Polytechnic University, Faculty of Humanities and Social Science, Macao, China; 2 Zhejiang Normal University, Xingzhi College, Jinhua, China

**Keywords:** HIV disclosure obligation, Privacy rights, HIV stigma, PLHIV, China

## Abstract

**Background:**

In 2021, a Chinese court, based on the newly enacted *Civil Code*, first revoked a marriage license due to the spouse’s failure to disclose their HIV infection before the marriage. This landmark case ignited a fresh debate on whether people living with HIV (PLHIV) have a legal duty to inform their spouses and sexual partners. Advances in medicine have partially isolated HIV transmission from sexual contact, extending the legal basis for the obligation to disclose beyond disease prevention. This study investigates some possibly unforeseen challenges for PLHIV in China to fulfill this duty, and the outcomes of their decisions in light of the government’s goal to promote health.

**Methods:**

This study aims to provide a detailed examination of the legal provisions and practices concerning partner notification among PLHIV in China. A mixed-methods research approach was employed between 2019 and 2020, combining questionnaire surveys, in-depth interviews, and participatory observations. A total of 433 valid responses were obtained through a questionnaire posted on a Chinese online platform for PLHIV. Following the collection and random coding of the questionnaire data, 40 individuals living with HIV were selected for in-depth interviews. Subsequently, a six-month field investigation was conducted in *Guan ai jia yuan* (Caring Home) in Jinhua City to further explore this issue.

**Results:**

A considerable proportion of PLHIV exhibit a high rate of disclosure to their spouses (nearly 80%). In the context of sexual partners, 56% of PLHIV stated that their sexual partners were aware of their HIV infection. Whether married PLHIV disclosing to their spouses or unmarried/divorced PLHIV disclosing to sexual partners, however, a substantial majority expressed apprehension about the potential disruption to their relationships that the disclosure might cause. The sole exception was observed among married PLHIV in extramarital relationships who demonstrated a slightly diminished level of concern in this context. Reasons for non-disclosure predominantly included undetectable viral load and the adoption of protective measures.

**Discussion:**

This study reveals that a prevailing “HIV stigma” hinders PLHIV from voluntarily fulfilling the disclosure duties bestowed by Article 38 of the Regulations on the Prevention and Control of HIV/AIDS, and the unclear legal provisions of the new Civil Code play a significant role in this regard. Addressing this issue necessitates not only increasing societal tolerance toward PLHIV and reducing instances of social exclusion but also shifting the legal basis of disclosure duties from disease prevention to rights and obligations within the legal relationships of the parties involved. When it comes to the recipients of disclosure, for instance, it is crucial to differentiate between spouses and sexual partners. As for PLHIV failing to fulfill their disclosure duties, apart from interventions involving indirect notifications, the addition of further legal responsibilities may not be advisable. Intentional transmission actions, on the other hand, should still be subject to severe penalties.

*Clinical trial number*: Not applicable.

## Introduction

Due to the distinctive nature of HIV transmission, issues related to the sexual behaviors, marriages, and reproductive rights of PLHIV have sparked extensive societal debate [1, 2]. Numerous studies have also conducted corresponding analyses of practical situations [3, 4]. However, given the legal concerns such as spousal rights and privacy rights, the encouragement of partner notification faces various specific legal and social challenges [5, 6]. In response, some countries have made proactive attempts to enforce or at least encourage HIV notification to sexual partners [7–9], and China is no exception [10, 11]. This study aims to provide a detailed examination of the legal provisions and practices concerning partner notification among PLHIV in China. Through empirical research, it seeks to uncover the multifaceted factors and challenges underlying this legal disclosure duty and recommend improvements.

Compared to the former Marriage Law of the People’s Republic of China,^1^ the 2021 Civil Code of the People’s Republic of China (China’s Civil Code)^2^ has changed the legal effect of marriage involving a party with a serious illness. The previous categorization of such marriages as invalid has been revised to make them revocable under the China’s Civil Code [12]. In other words, the recognition of the legal effect of marriages arising from serious illness^3^ has shifted from being determined by law to becoming a claim-right of the parties involved. For PLHIV intending to enter into marriage, this provision not only safeguards their autonomy in marriage but also imposes higher requirements on PLHIV to fulfill disclosure obligations before marriage. It represents a further refinement of the disclosure obligations under the Regulations on the Prevention and Control of HIV/AIDS.^4^ Analyzing judicial precedents in China can stimulate further consideration of legal provisions [13]. On January 4, 2021, the Minhang Court in Shanghai issued a the first decision apply the China’s new *Civil Code*^5^ in annul a marriage [14].

In this case, the plaintiff, Ms. Li, and the defendant, Mr. Jiang, met through a mutual acquaintance and quickly established a romantic relationship. After becoming engaged, they began living together. In June 2020, Ms. Li became pregnant, and the two registered their marriage. After marrying, Mr. Jiang confessed to his wife that he had been living with HIV for several years and was on long-term antiretroviral medication. Although Mr. Jiang insisted that his viral load had become undetectable after antiretroviral treatment, rendering the possibility of transmitting the virus to Ms. Li and their unborn child extremely low, and although it was later confirmed that Ms. Li had not been infected, Ms. Li could not accept the situation. Despite their previously strong emotional bond, Ms. Li, after much inner turmoil and contemplation, decided to terminate her pregnancy and sued for annulment of the marriage in the Minhang Court in Shanghai. After a thorough investigation, on January 4, 2021, the court, based on China’s 2021 *Civil Code,* art. 1053, ruled to annul the marriage between the plaintiff and defendant.

Being the first ruling since the enactment of Article 1053 of China’s new *Civil Code*, this decision garnered widespread attention from various sectors of society. Along with this, a series of questions regarding the ruling have emerged. Do PLHIV who were not diagnosed before marriage still have a duty to disclose to their spouses after marriage? It is evident that PLHIV continue to face many practical differences and challenges when it comes to the duty to inform their partners [15]. This landmark case highlights several crucial questions. First, do PLHIV have an obligation to disclose their condition to their partners? How should the potential intersections between the time of infection, diagnosis, and marriage be considered? Secondly, there is an essential distinction between sexual partners and spouses. How should specific legal regulations based on the risk of HIV transmission address issues on the periphery, such as non-marital sexual activity or asexual marriages? Thirdly, is there a need for state intervention regarding the duty of PLHIV to inform their partners? Even if relevant authorities possess the power to actively intervene at the legal level, is the exercise of such authority a matter of “ought to” or “can”? Moreover, could the act of disclosure within marital and family relationships have disproportionately negative consequences on the personal lives of the PLHIV? These questions demand further in-depth analysis and discussion even after the Minhang Court’s decision of this case.

## Method

### Questionnaire surveys, in-depth interviews, and participatory observations

By comparing the real-life practices of PLHIV with the legislative regulations, we can gain a better understanding of the practical issues behind the operation of a legal duty to disclose [16]. Ultimately, this may lead to recommendations for further improvements in legislation and enforcement. In light of this prospect, this study adopts a mixed-methods research approach, which includes questionnaire surveys, in-depth interviews, and participatory observations, to investigate the PLHIV community on this subject. Leveraging China’s largest PLHIV support organization, the *Bai Hua Lin National Alliance*,^6^ and the social organization *Guan ai jia yuan* (Caring Home) in Jinhua City, we conducted in-depth research within the PLHIV community. This approach allows us to gain insights into the numerous subjective and objective factors that PLHIV (and thus, the law as well) need to consider when facing the duty to disclose.

During the questionnaire phase, to observe the social practices of PLHIV disclosure obligations in China under the current legal regulations from a more comprehensive perspective, we opted to avoid collecting samples from a single or specific region. To ensure the comprehensiveness and representativeness of the sample collection, we collaborated with Bai Hua Lin National Alliance, the largest PLHIV mutual aid platform in China. First, this study utilized an online questionnaire format, posted on the largest Chinese platform for PLHIV support, the *Bai Hua Lin National Alliance*. In the questionnaire submission system, the informed consent of the participants was explicitly obtained anonymously. The questionnaire was prepared and selected through informal discussion within the research unit and through comprehensive research. The questionnaire primarily aimed to address the following aspects of PLHIV disclosing their HIV status to spouses and sexual partners:

1. The marital and sexual relationship status of the participants, along with their antiretroviral treatment details;
2. The extent of knowledge regarding the participant’s HIV status among their spouses and sexual partners, as well as the channels through which these acquired this knowledge;
3. The factors influencing the participant’s choices, regardless of whether they disclosed their HIV status;
4. The participants’ views and attitudes toward the current legal regulations in China from a normative perspective.

After analyzing and randomly numbering the collected questionnaire data, we reached out to 132 individuals who expressed a willingness to participate in further interviews. Ultimately, in-depth interviews were conducted with 40 of these individuals. Subsequently, we carried out a field investigation related to this issue for approximately six months at *Guan ai jia yuan* (Caring Home) in Jinhua City. The reasons for selecting Caring Home in City Jinhua, Province Zhejiang, as the point of observation for this research are as follows: (1) City Jinhua in Province Zhejiang is not traditionally considered a high-prevalence area for HIV/AIDS in the country; the economic development level in City Jinhua is considered moderate within Province Zhejiang and nationally. The study aimed to refrain from investigating individuals with unique characteristics or experiences within the national PLHIV population, in order to enhance the representativeness of the research. (2) The author received strong support from the leadership and staff of the Infectious Disease Division at the Jinhua City CDC. Through extensive coordination efforts, the author was able to visit the compassion clinic at the designated HIV hospital in City Jinhua twice a week as a volunteer. (3) The author received significant assistance from the primary responsible person at the Caring Home organization in City Jinhua. Thus, the author had the opportunity to participate in leisure and recreational activities organized by Caring Home for PLHIV. This mode of contact minimized communication barriers between the researcher and the research subjects. In 2020, the author visited the Caring Clinic as a volunteer when it was open (all day on Tuesdays and Sunday mornings) each week. However, because the Caring Clinic is full of people coming and going during its working hours, the author would usually document representative instances at the end of his day’s work and, if he found a specific PLHIV who was more relevant to this study, the author would make arrangements for a separate interview with the person concerned, subject to their consent.

### The effects of precedents on the 2021 Civil Code in China

Analyzing China’s new legal regulations with precedent cases can help us explore from multiple perspectives the various underlying attitudes of PLHIV toward their duty to disclose. Comparing the Minhang Court case, *Li v. Jiang* (2021) with cases under China’s earlier *Marriage Law* (2001) reveals significant change in determining the validity of marriage where major illness is involved. First, under the new law, marital relationships involving one party with a major illness are no longer flatly invalid but may be deemed revocable.^7^ Secondly, the 2021 *Civil Code* introduces a pre-marriage duty of disclosure concerning major illnesses. The new provisions, however, do not appear to be universally applicable to cases where major illnesses were not disclosed *before* marriage, as under the previous law [17].

For disputes concerning marriages that fall under the former *Marriage Law of the People’s Republic of China*, there still exists a legal hierarchy conflict under non-retroactivity, Article 7 of this law, stipulates: “Marriage is prohibited in the following situations: … (2) suffering from a disease recognized by medical science as unfit for marriage.” A bit more research is required to determine the meaning of “disease recognized as unfit for marriage.” *The Maternal and Child Health Law*, art. 8, provides: *“*Pre-marital medical examination includes the examination of the following diseases: (1) severe hereditary diseases; (2) designated infectious diseases; (3) relevant mental illnesses*.*” Further, Article 38 defines *“*designated infectious diseases” as those listed in the “*Infectious Disease Prevention and Control Law of the People’s Republic of China*,” including AIDS, syphilis, gonorrhea, leprosy, and other infectious diseases recognized by medical science as affecting marriage and reproduction. Therefore, despite the protection of the marital rights of PLHIV being granted under the *2006 Regulations on the Prevention and Control of HIV/AIDS*, decisions declaring marriages as valid or invalid based on non-disclosure of an illness still challenge the courts. One example finding invalidity of the marriage was the decision of the Xiaoshan Court below:

[A]ccording to the *Marriage Law of the People’s Republic of China*, ‘If, before marriage, one party is afflicted with a disease that is recognized by medical science as one that makes marriage inadvisable, and the disease is not yet cured after marriage,’ this constitutes one of the situations of invalid marriage. In this case, the defendant was confirmed as a PLHIV during the pre-marital examination on the day of marriage registration. According to the provisions of the *Maternal and Child Health Law of the People’s Republic of China* and the *Infectious Disease Prevention and Control Law of the People’s Republic of China,* HIV is considered a disease that makes marriage inadvisable from a medical perspective. Therefore, the plaintiff’s lawsuit request, backed by sufficient and law-compliant evidence, is supported by this court. The court declares the marriage between Mr. Luo and Ms. Xu invalid^8^ (2014)*.*

However, According to Article 5 of the *Marriage Registration Regulations*, promulgated and implemented in 2003, “Mainland residents applying for marriage registration should provide the following documents and proof materials: (1) Their household registration booklet and identity card; (2) A signed declaration stating that they are not married and have no direct consanguinity or collateral consanguinity within three generations with the other party involved.” This court understood, by omission, that pre-marital health checks are no longer a prerequisite for marriage registration. Thus China’s marriage inspection system for illness had shifted from mandatory to voluntary. Whether PLHIV preparing to enter into marriage need be aware of their HIV infection status becomes a matter of voluntary choice. Due to the relatively long incubation period of HIV, if a person is unaware of their HIV infection status at the time of marriage but later becomes aware, this flexibility is welcome. Similarly, in cases where a person is infected with HIV after marriage but no longer engaging in sexual activity with their spouse, disclosing their HIV status is no longer mandatory. (*The Regulations on the Prevention and Control of HIV/AIDS* explicitly state that PLHIV are obligated to disclose their status to individuals with whom they engage in sexual activities,^9^ excluding those in a marital status without sexual activity). However, for this flexibility, the law provides no guidelines. If one party infected with HIV or becomes aware of their infection status after entering into marriage, two possible scenarios may arise. One scenario is the already discussed choice of the PLHIV to disclose, with all the potential uncertainties that decision entails. Another scenario, however, is when the PLHIV refuses to fulfill their duty to disclose in order to “maintain the marriage.” In a divorce dispute judgment based on such a scenario, the Lengshuitan court found that:

The plaintiff, Mr. Zhang, and the defendant, Ms. Qin, were first in a romantic relationship, then cohabited without being married, and later voluntarily married and had a child. They had a stable emotional foundation. After the defendant, Ms. Qin, was diagnosed with HIV, she concealed her condition from the plaintiff, Mr. Zhang, and insisted on having a child to maintain the marriage. Fortunately, the child did not contract the virus. While the defendant’s actions involved wrongdoing [failure to disclose], her motive was to preserve the marriage. Under these circumstances, the court found discrimination against the PLHIV was unwarranted. The court deemed rather that the plaintiff and her husband, must offer greater understanding and support to one another. For the sake of societal stability, family harmony, and the healthy upbringing of their child, divorce shall not be allowed for the plaintiff and the defendant^10^ (2015).

In another scenario, when a PLHIV voluntarily disclosed their HIV status to their spouse after marrying, the court weighed more heavily the claimed or at least potential breakdown of their relationship based on the old Marriage Law. By contrast with the Lengshuitan court above, the Wuhou court ruled:

According to Article 3, Section 2 of the *People’s Republic of China Law on the Prevention and Treatment of Infectious Diseases*, “HIV/AIDS belongs to Class B infectious diseases.” Since diseases of this class affect marriage and reproduction, it is not appropriate for the plaintiff and the defendant to live together. Therefore, the court supports the plaintiff, Mr. Li’s, request for a divorce. As for the defendant, Ms. Tian’s argument that, despite having HIV, she does not agree to divorce because the plaintiff indicated acceptance, this defense is not accepted due to a lack of legal and factual basis^11^ (2014).

The first case above show that the failure of a PLHIV to inform their spouse need not always lead to a change in the legal status of their marriage. Conversely, however, the act of disclosure may result in a change in the marital relationship. From the perspective of the principles of marriage law,^12^ the court does not appear to have the authority to substantially interfere with the autonomy of a marriage based solely on HIV infection. Yet, the court seems also unable or unwilling to hinder one party’s desire to discontinue the marriage upon learning of the other party’s HIV infection, despite near harmless indications. These differing outcomes raise the concern that when PLHIV fulfill their duty to inform their spouses, even if the court does not substantially intervene in the legal status of their marriage, they may still face potential “adverse consequences” based on their voluntary decisions. This study aims to delve deeper into the legal as well as practical aspects of HIV disclosure, building upon the research findings, to observe the subjective attitudes of PLHIV toward their duty to inform and the underlying real-world issues.

## Results

### Basic characteristics of the study population

The survey collected a total of 496 online questionnaires, of which 433 were deemed valid, resulting in an effectiveness rate of 72.2%. (This study implemented stringent screening mechanisms during the sample selection process, which included the exclusion of questionnaires with a completion time of fewer than 5 min and incomplete informed consent information, among other criteria). Of the respondents, 96.3% identified as male, 3.2% as female, and 0.5% as transgender individuals. The higher proportion of male respondents is closely associated with the Men who have Sex with Men (MSM) population [18], where anal sex is a sexual practice that can increase the risk of HIV transmission [19]. Regarding age distribution, the majority of the participants in the survey fell within the young and middle-aged groups, with 41.3% of respondents aged 31–40, followed by 36.5% aged 21–30, 16.6% aged 41–50, and 5.5% aged 51–60. In terms of marital status, the most common category was unmarried (never married), accounting for 66.1% of the respondents. The percentages of married and divorced individuals were lower, at 25.3% and 8.6%, respectively. It is worth noting that 99.1% of the surveyed individuals were currently taking antiretroviral medications, and 88.5% of the respondents had viral load values less than 200 copies/ml (including undetectable levels) based on viral load testing.

### Analysis of PLHIV disclosure to spouses and sexual partners

#### Disclosure among married PLHIV to spouses and sexual partners

Within the group of married PLHIV who participated in the questionnaire, 44.4% reported that they were still engaging in sexual relationships with their spouses. Notably, 79.2% of the married PLHIV indicated that their spouses were aware of their HIV status. Of these, a remarkable 95% of the surveyed individuals proactively fulfilled their disclosure obligation. Of the 20.8% who had not informed their spouses about their infection, some expressed deep concern about the potential impact on their relationship of disclosing to their spouses, but a substantial 90.9% of the PLHIV believed they had already taken protective measures during sexual intercourse. Furthermore, a majority (54.6%) of all married PLHIV reported that their viral load had become undetectable, and therefore they considered themselves incapable of transmitting the virus based on the “Undetectable = Untransmittable (U = U)” principle.

A minority of married PLHIV (39%) reported having stable sexual partners other than their spouses, and among them, 56% of the married individuals stated that these sexual partners were aware of their HIV status. Among this subgroup, a substantial 92.9% of married PLHIV had voluntarily disclosed their infection to their sexual partners. Among those whose sexual partners were unaware of their HIV status, 72.7% of the PLHIV based their decision not to disclose on the adoption of protective measures during sexual encounters. Furthermore, 81.8% believed their viral load was undetectable, rendering them non-infectious according to the U = U concept. As for concerns about potential strain in their relationship with sexual partners, this was relatively low, with only 36.4%.

Insights from the interviews revealed that many married PLHIV displayed varying tendencies when it came to disclosing their status to spouses and sexual partners. These choices were often more rooted in the assessment of maintaining relationship rather than the infectiousness of HIV.

I realized my sexual orientation was different from most boys when I was in my teens. Although I had thoughts about living with a same-sex partner, societal pressures and personal reasons forced me to use marriage as a disguise. Life with my wife after marriage wasn’t exactly blissful, but it was bearable. We had a child in the second year, which made our parents very happy. They helped us take care of our child and manage our lives. At this point, I suddenly felt like my mission was complete, and those thoughts started to stir again. So, I began to arrange encounters with men through online chat rooms. I was afraid of affecting my family, so I didn’t dare to establish long-term relationships with them. Perhaps this is also why I haven’t told my wife since I was infected (Participant #48)

#### Disclosure among unmarried PLHIV to their sexual partners

30.7% of unmarried PLHIV reported having stable sexual partners, and 66.3% of these individuals indicated that their sexual partners were aware of their HIV status. Regarding the disclosure process, a significant majority of them, specifically 96.7% took the initiative to inform their sexual partners about their HIV status. In cases where sexual partners remained unaware of the respondents’ HIV status, 90% of those surveyed believed that their viral load was undetectable and considered there to be no risk of transmission within the U = U concept. Additionally, 73.3% reported using protective measures during sexual activities. 76.7% expressed concerns about the potential breakdown of their relationships with sexual partners if they disclosed their HIV status.

Unlike spouses with established legal relationships, unmarried PLHIV often have distinctions between stable and non-stable sexual partners. Unstable relationships leads to a reduced incentive for disclosure, particularly in cases involving casual or occasional sexual encounters, as indicated by the following interviewee.

I currently don’t have any friends (regular male partners)... but can easily meet people online. Who asks for names and stuff nowadays, that’s so weird, right? Even if you ask, they won’t tell you. Just send photos; that’s enough. Plus, most have ‘read and disappear,’ meaning the photos vanish a few seconds after you view them. Usually, after the meetup, there’s no further contact unless you come across someone special. When I arrange a casual encounter, it’s just for that, no need to chat about this and that. If you don’t want to hook up, just say so. Tell someone in advance that I have this (HIV)? Then, what’s the point of meeting up? But I’ll definitely use protection. It’s not just to avoid passing it on to someone else; I’m also afraid of catching syphilis, genital warts, hepatitis B, hepatitis C, and all those other diseases. (Participant #429)

#### Disclosure among divorced PLHIV to former spouses and sexual partners

Among divorced PLHIV, approximately 31.6% had stable sexual partners, and 83.3% of these PLHIV stated that their sexual partners were aware of their HIV status. Notably, all of these PLHIV (100%) voluntarily fulfilled their duty to disclose their HIV status to their sexual partners. Of those whose sexual partners were unaware of their infection, all of the surveyed individuals (100%) expressed concerns about potential relationship breakdown. Approximately 50% either practiced safe sex or believed there was no risk of transmission due to undetectable viral loads. Among this group, 79% stated that they were diagnosed with HIV after getting married. Among those who were diagnosed after marriage, 63.3% of PLHIV had proactively disclosed their HIV status to their spouses. Of those who didn’t disclose, 27.3% claimed that they had confirmed through other means that their spouses were uninfected. Furthermore, 18.2% of those who did not disclose cited concerns about relationship breakdown, self-perceived non-transmission risk, and taking protective measures, respectively. Among PLHIV who were diagnosed with HIV before marriage (21% of cases), 44.4% had proactively disclosed their HIV status to their spouses. Among the remaining 55.6% surveyed, 75% stated that they did not disclose because they were concerned about the breakdown of their family relationships. In addition, they maintained that they were no longer under the obligation to disclose due to their current divorced status. Another 25% of this group believed that they had no risk of transmission either due to practicing safe sex or because their viral loads were undetectable.

It can also be observed from the interviews that some unpleasant marital experiences further discouraged divorced PLHIV from disclosing their HIV status.

I was diagnosed with HIV after it was discovered during a minor surgery. After finding out about my infection, I went for tests and started taking medication. At the time, my wife accompanied me to the clinic, but later, her attitude changed drastically. She often used harsh words and even asked me to kill myself. She disclosed my condition to her close friends and even told our son. If I had known it would turn out like this, I would never have disclosed my status [to my wife]. (Participant #378)

### PLHIV Subjective willingness regarding disclosure obligation

Overall, the survey showed that 61.2% of PLHIV believe they have a duty to disclose their status to their spouses and sexual partners. Among them, 87.4% believe it stems from the fidelity duty within a marriage, while 79% see this obligation as a means to safeguard the health rights of others. Among the 38.8% of PLHIV who do not feel obligated to disclose their status to spouses and sexual partners, 81.9% are concerned about the potential serious adverse effects of personal privacy being breached, while 84.6% believe that alternative measures can be taken to prevent the transmission of the virus.

What is noteworthy is that with the increasing promotion of the U = U(Undetectable = Untransmittable) concept in society [20] and the widespread adoption of safe sex practices [21], particularly with supporting evidence, contagiousness is not a significant influencing factor when considering whether to disclose one’s HIV status. We encountered numerous instances of this during interviews:

I noticed a rash on my body, and when I used an HIV test kit, I was almost certain that I had contracted HIV. After some time, I went to a disease control center for testing, and the infection was confirmed. I went for testing together with my wife because we have been sexually active in our marriage, and I was concerned about her getting infected. Fortunately, my wife is healthy, probably because we have been using protection. (Participant #48)

Another instance of hesitancy to disclose due to societal reason: a volunteer once shared the situation of a PLHIV during an interview:

He (heterosexual) was already infected with HIV before getting married. He mentioned that his family didn’t know about it, and as he got older, there was more pressure to get married and have children. Later, he met a woman (who was not infected with HIV), and they got along well, considering marriage. He was too afraid to tell her because he thought that revealing his HIV status might jeopardize the marriage prospects. Besides, they come from a small town, and if he disclosed his status, he was worried about how he would be treated there. They later decided to have children. At that time, he had been taking medication for three years, and his CD4 count was high, with an undetectable viral load. He consulted doctors to ask if it was possible to have a child. The doctor would probably recommend that the woman also take pre-exposure prophylaxis (PrEP), but he didn’t want to tell her about his HIV status. After asking several places, the general response was that if the viral load cannot be detected, there’s a 90% chance that everything is fine. However, they recommend taking PrEP. In the end, he chose not to disclose his status. Now, their child is almost a year old, and everything is fine. (Participant #203)

Additionally, the actual experience of societal stigma after disclosure, especially the isolation from family support, can have a seriously negative, even life-altering effect on a PLHIV:

I found out about my infection when I had a fever and went for a blood test. Shortly after the test, the hospital called my family without notifying me. My family, upon learning about my infection, distanced themselves from me, and I had to become self-reliant. I dropped out of college and started working as a waiter. My friends, once they found out about my situation, started treating me coldly or just ignored me, so I deleted them from my life. Now, I’ve gradually grown accustomed to being on my own. I don’t really feel like looking for a partner anymore, and I’m not sure how long I can keep going. (Participant #148)

## Summary move to conclusion

According to the research (as shown in Table 1), it can be observed that the majority of PLHIV, whether married or with a steady sexual partner, inform their spouse or partner of their HIV infection. Of these, a greater percent of married partners inform their spouses (nearly 80%) than those with regular sexual partners (66%). The unmarried PLHIV disclosure might be relatively lower compared to a legally wedded partner, because the level of commitment between sexual partners may be somewhat more fragile.

**Table 1:** Consolidated summary of marital status and partner communication

	Marital status						Total	
	Married		Unmarried		Divorced			
	Count	Percentage	Count	Percentage	Count	Percentage	Count	Percentage
*Whether spouse is informed*								
Yes	87	79.2			22	59.5	109	87.1
No	23	20.8			15	40.5	38	25.9
Total	110	75			37	25	147	100
*Whether regular sexual partners are informed*								
Yes	24	56	58	66	9	82	91	64
No	19	44	30	34	2	18	51	36
Total	43	30.2	88	62	11	7.8	142	100
*Whether there is a (another) steady sexual partner*								
Yes	43	39	88	30.7	11	31.6	142	32.8
No	67	61	198	69.3	26	68.4	291	67.2
Total	110	25.3	286	66.1	37	8.6	433	100

Case histories indicate that reasons for non-disclosure are due to concern that disclosing their status might lead to relationship breakdown, as well as fears of offending other family members (parents), or else based on the U = U concept, the PLHIV believes they are taking adequate precautions (antiretroviral medication, contraceptives, or even abstinence).

In terms of how they became aware of their partners’ knowledge, over 90% of participants who had informed their spouses or sexual partners had done so voluntarily. This emphasizes the significance of voluntary disclosure as the primary means of fulfilling the obligation to inform. If a PLHIV is resistant to this obligation, external intervention may not necessarily yield positive results. It is important to note that divorced individuals have a slightly lower rate of voluntarily informing their former spouses during the duration of the marriage relationship, which is related to their marital status at the time of diagnosis. The level of concern about disclosure among married individuals regarding extramarital sexual partners was slightly lower than for PLHIV spouses, again due to the greater level of commitment in marriage.

## Discussion

### Challenges of the legal obligation: when and whether to disclose

Article 38 of the *Regulations on the Prevention and Control of HIV/AIDS* requires PLHIV to disclose the fact of their infection or illness to those with whom they have sex.^13^ In some provinces, additional regulations have been introduced to specify the term “spouse” to strengthen the somewhat vague legal concept of “those with whom they have sex.”^14^ However, it is important to note that these two concepts clearly refer to different situations, and the circumstances of PLHIV can vary widely. It is worth mentioning that sexual activity and marriage are not as tightly connected in China as one might think [22]. The survey conducted in this research has revealed that, even under the condition where China’s Civil Code stipulates monogamous^15^ marriage, 39% of married PLHIV have more than one steady extramarital sexual partner. During interviews, we discovered that this phenomenon is related to traditional views on heterosexual marriage in China [23]. PLHIV may, under societal and familial pressures, enter into “marriages of convenience” or “sexless marriages” with individuals for whom they do not have mutual romantic feelings. Furthermore, in “marriages of convenience” or “sexless marriages,” spouses may not necessarily engage in sexual activity [24]. Same-sex marriage is not yet legalized in China [25], so MSM, considered one of the “high-risk groups” for HIV, often enter into “marriages of convenience” with female same-gender (FSG) partners due to social pressures [26]. In such marital relationships, sexual relations may not even exist between the spouses, so they cannot be considered sexual partners. The objective existence of such phenomena contradicts the original intention of requiring PLHIV to fulfill their disclosure obligations from both legal and public health prevention and control perspectives.

It is thus evident that trying to merge the concept of “spouse” with “sexual partner” in terms of disclosure obligations has significant flaws. Why then do some regions choose to use “spouse” in place of “sexual partner” in setting disclosure obligations? Compared to sexual partnerships, there are clear rights and obligations within marriage (such as cohabitation obligations) [27]. Thus, in planning to wed a PLHIV, their potential spouse deserves the right to *pre-marital disclosure*,^16^ and the autonomy to decide whether or not to enter into the marriage after learning of their partner’s condition. that is to say, be allowed.^17^

However, life is not always that simple. Some PLHIV may not have been diagnosed until after marriage, or may have been infected for some time without knowing it, which complicates the matter of whether the disclosure obligation applies to their spouses or sexual partners. Currently, Chinese law simply lists sexual partners and spouses, without further distinguishing between the two.^18^ Real-life situations can be far more complex than what the law dictates, however. The blurred definitions of “spouse” and “sexual partner” undeniably pose a challenge to applying the law [28], which may require further judicial, if not legislative distinction between the two. Additionally, beyond the differences between the concepts of spouses and sexual partners, for any individual whose sexual partners are occasional and not fixed, it may be difficult for both the PLHIV and institutions such as the CDC to provide accurate reports on disclosure [29].

As for casual sexual encounters and sex work, it is challenging for parties other than the individuals involved to discover the sexual relationships that may have infected the PLHIV or the individuals who should be notified [30]. Even if the existence of such sexual encounters is known, based on the privacy and anonymity features that certain policies require in apps and software [31], it is equally difficult for PLHIV to provide accurate disclosure. Moreover, in areas where local regulations grant “mandatory disclosure rights” to the CDC, healthcare institutions, etc., as the PLHIV lack precise locating technologies similar to criminal investigations, this so-called “mandatory disclosure” often ends up being limited to the legally defined “spouses” [32]. This clearly results in a disconnect between application of the law, and its intended disease prevention and control.

It is important to note that from a medical perspective, engaging in sexual activity as a PLHIV does not necessarily lead to infection. It depends on the manner of sexual activity, the use of protective measures, and the viral load of the PLHIV [33]. Undetectable = Untransmittable (U = U) medically implies that, for the PLHIV who has undergone antiviral treatment as prescribed, if their HIV viral load remains undetectable for *over* 6 months, the risk of transmitting the HIV virus to their HIV-negative partners through sexual activity is not likely to result in transmission. In other words, undetectable viral load equals non-infectiousness [34]. For PLHIV with undetectable viral loads, unprotected sexual activity is not likely to result in transmission. So, the question arises whether legal prerequisites are needed for PLHIV to disclose their status to “sexual partners,”^19^ or whether it might not be better to first define the more complex distinctions within the concept of “PLHIV” from a medical standpoint.

As often emphasized in HIV prevention campaigns, taking protective measures during sexual activity (such as correct condom use) can significantly reduce the possibility of HIV transmission [35]. For persons who test positive, the failure to disclose their status is almost never due to intentional transmission. During interviews, we found that most PLHIV reluctance to disclose their status is hardly driven by mens rea but rather considerations about societal acceptance, concerns about personal privacy, and family harmony. For this reason, PLHIV who have taken protective measures before sexual activity may cite above reasons as a primary reason for not fulfilling their disclosure obligation, and the law has yet to make explicit provisions permitting this exception. There is ample room for discussion about whether sexual activities with very low risk of transmission [36], such as oral sex, should also fall within the scope of a potential disclosure waiver.

During interviews, it was evident that for PLHIV who are married or have stable partners, their reluctance to fulfill their disclosure obligation is often rooted in fear of relationship breakdown. If a relationship deteriorates, it can lead to the disclosure of the PLHIV status by the informed party, and in some cases, it may even be used to threaten the PLHIV. While Article 39 of the *Regulations on the Prevention and Control of HIV/AIDS* imposes a duty of confidentiality on third parties, it lacks punitive provisions for legal consequences.^20^Through interviews, it is evident that for married PLHIV, given the close-knit nature of family relationships, disclosure between spouses often extends to the entire network of friends and relatives centered around the family. This can have a profoundly detrimental impact on the PLHIV. If the counterparty to the disclosure obligation fails to uphold the duty of confidentiality, it can significantly affect the PLHIV life, with no ill effects to the counterparty particularly in rural areas, the knowledge of both families can sometimes extend to the entire village. In such cases, the privacy of PLHIV is challenging, if not impossible to restore. Therefore, legislative measures need to provide clear safeguards for PLHIV privacy while defining the disclosure obligation.

Furthermore, among the PLHIV interviewed, most expressed how challenging it becomes to find suitable partners or spouses after their HIV diagnosis. Typically, PLHIV experience a shrinking social circle, and they themselves become sensitive, often avoiding or fearing social interactions. In other words, the stigma associated with HIV that looms around PLHIV is another major reason for their reluctance to fulfill their disclosure obligation. This external pressure can even lead to some PLHIV experiencing a sense of despair. The phenomenon of self-stigmatization among PLHIV reflects a series of shortcomings in China’s public health institutions in addressing HIV-related stigma, thereby hindering PLHIV from fulfilling disclosure obligations as stipulated.

### Recommendations and suggestions

Throughout it recent history, HIV prevention and control have remained integral to global public health efforts [37]. The initial intention behind the establishment of the Article 38 legal obligation to disclose one’s HIV status was to curb the spread of the virus in China. However, due to the ongoing need for enhanced public education and awareness about HIV at the societal level [38], this disclosure obligation is extremely complex in practice. The prevailing “stigma surrounding HIV” remains widespread [39], and the roots of HIV-related anxiety are complex [40, 41]. Refraining from risky behavior and ensuring one’s protection is indeed the most effective means of prevention.

The Joint United Nations Programme on HIV/AIDS(UNAIDS), the International AIDS Society, the World Health Organization, and other authoritative international organizations have all endorsed the “U = U” medical standard mentioned earlier. They are committed to establishing the “U = U” concept as a crucial scientific consensus in the field of HIV/AIDS prevention and control [42]. For PLHIV, the widespread adoption of the “U = U” concept is particularly beneficial, as it can help reduce their internal anxieties and facilitate better living and social integration. Given these considerations, we believe that legal provisions should take the “U = U” medical concept as a premise. The establishment of disclosure obligations should not primarily depend on the consideration of HIV transmission risks. This is because, with the scientific use of protection measures such as condoms and the medical premise of “U = U,” sexual contact between PLHIV and others does not necessarily result in HIV transmission. Drawing on the experiences of HIV/AIDS prevention and control legislation from various regions in China, we propose that legal provisions related to disclosure obligations should be explored from the perspectives of both “spouses” and “sexual partners,” encompassing both rights and responsibilities.

Non-marital sexual partners do not have clearly defined rights and obligations between them. To impose a disclosure obligation on them, the only dimension of reasoning is the risk of disease transmission. However, HIV, as a disease, can be effectively prevented by using measures like condoms [43]. With the exception of a small number of minors (which would involve criminal issues, as explained later), most adults engaged in non-marital sexual activities are considered legally competent individuals. They should have a basic understanding of risk prevention and be responsible for their own actions. In other words, since complete information exchange is impossible, any legal entities involved in non-marital sexual activities should proactively bear the risk of disease transmission and take appropriate precautions. From the perspective of consensual sexual relationships, these interactions are within the realm of personal privacy, which should be respected by public authorities. Health authorities, as administrative bodies, should focus more on the accessibility of disease prevention. Of course, from an ethical standpoint, we still encourage PLHIV to voluntarily disclose their status to their sexual partners.

Furthermore, our research has found, for certain social groups, their sexual partners are not fixed and encounters are occasional. In such casual sexual relationships, PLHIV face practical challenges in terms of disclosure. Firstly, since these relationships are occasional, the information available to PLHIV about their sexual partners is often incomplete, and these interactions may be limited to brief physical encounters. Consequently, PLHIV may not have the incentive or willingness to disclose under these circumstances. Secondly, sexual relationships are inherently private, regardless of the potential for health authorities’ intervention [44]. Behind this lies the risk of intruding on the privacy of PLHIV sexual partners. As a result, for these types of relationships, where encounters are sporadic and partners may have limited interaction beyond the sexual act, enforcing a mandatory disclosure obligation for PLHIV becomes impractical and raises concerns about privacy infringement on the part of their partners.

However, unlike casual sexual partners, legal spouses in a marriage have a defined set of legal rights and responsibilities. Being fully informed is a prerequisite for entering into a marital relationship.^21^ Post-marriage, spouses have a range of legal rights and obligations, including fidelity obligations.^22^ Firstly, when entering into a legal marriage, both parties should engage in a comprehensive disclosure process to ensure that both individuals are fully informed about each other’s status. This is necessary to safeguard the marital relationship and prevent the risk of invalidity or annulment (as stated in Articles 1053 and 1054 of China’s Civil Code). Thus, PLHIV who have been diagnosed before marriage should proactively disclose their infection status to their prospective spouses. Secondly, within a legal marriage, spouses have various rights and responsibilities. The fidelity obligation means that the sexual relationships between spouses should be exclusive during the marriage. The cohabitation obligation grants either spouse the right to request the other to live together during the marriage.^23^ These rights and responsibilities form the legal basis for PLHIV to disclose their infection status to their spouses. While marriage as a social institution does not inherently lead to disease transmission, and HIV, as discussed earlier, is preventable, legal duties based on the rights and obligations of spouses should still apply, even in objectively non-sexual marriages. This is to ensure that the legal integrity of the institution of marriage is upheld, protecting the interests and rights of both spouses. Unlike sexual partners, spouses should, in principle, not engage in extramarital sexual activities. Therefore, they cannot be required to practice self-risk prevention within the marriage, as non-spousal sexual partners might.

It should be noted that the above legal analysis of the disclosure obligations from the perspective of spousal fidelity is clearly carried out from the viewpoint of the legal system, which may, to some extent, be in tension with the public health perspective. Continuing the analysis from the previous section, spousal rights in China constitute an organic whole, including cohabitation obligations, fidelity obligations, and support obligations. Therefore, although the act of disclosure may potentially have some adverse consequences for PLHIV beyond the legal realm, at least from the perspective of the relative nature of rights and obligations, it still holds ground. This also aligns with the societal morality and the orientation of public health. However, if we push the perspective forward, the law often comes with state coercion, meaning that as a legal disclosure obligation, setting responsibilities or even enforcing them is not unreasonable. But at this point, it seems to deviate from the direction of public health. In the preceding text, we extensively argued the differences in individual performance of disclosure obligations in legal practice, such as demonstrating in detail the various motivations for PLHIV reluctance to fulfill disclosure obligations in the research results. Therefore, the enforcement of disclosure obligations may even lead to individuals evading HIV medical testing and treatment, which clearly contradicts the overarching goal of HIV prevention and treatment of “early detection and early treatment” [45]. In this sense, compared to the societal effectiveness that public health, emphasizing flexible governance, can achieve, the legal definiteness may serve better as a bottom-line obstacle.

Furthermore, since there is currently no vaccine or cure for HIV [46], and for the sake of maintaining social order, relevant Chinese authorities should strongly penalize the deliberate transmission of HIV. In this regard, the 2017 “Interpretation on Several Issues Concerning the Application of Law in Handling Criminal Cases of Organizing, Forcing, Luring, Sheltering, or Introducing Prostitution” issued by the Supreme People’s Court and the Supreme People’s Procuratorate in Article 12 stipulates that “if a person knowingly infects others with the HIV virus through sexual contact without taking preventive measures, they will be convicted and punished for intentional injury.”

However, it is essential to note that failing to disclose HIV status does not equate to intentional transmission, as the absence of disclosure does not inherently imply that PLHIV have mens rea to spread the virus to their spouse or sexual partner. The research in this article has revealed that many PLHIV are also victims themselves. After learning about their infection, they experience extreme fear and worry about accidentally transmitting the virus to others. Therefore, simply attributing “intentional transmission” to PLHIV who do not disclose their status is not entirely appropriate. Intentional transmission refers to the actions of a very small number of PLHIV who seek to transmit HIV to others from mens rea due to distorted mental states. Such behavior falls within the scope of “subjective extreme malice” regulated by criminal law. Therefore, the fundamental aim of the national legislation regarding intentional transmission is to establish a “bottom line”—while positively requiring PLHIV to actively disclose, it serves as a warning to those with intentional transmission. Those who spread the virus form mens rea will face severe penalties from the state.

## Conclusion

Considering the unique nature of AIDS as a presently incurable but treatable disease, whether PLHIV should bear the responsibility of disclosing their status to spouses and sexual partners requires a careful balance between public health safety and privacy rights, as well as consideration of the prerequisites, subjects, and methods of disclosure. Data analysis from the questionnaires and findings from interviews show that most PLHIV recognize the obligation to disclose to spouses and sexual partners based on the principles of safeguarding others’ right to health and the duty of fidelity within marital relationships. However, the statistical analysis of the intentions of PLHIV who refuse to disclose reveals that their reasons tend to be concentrated: firstly, concerns about the breakdown of intimate relationships, and secondly, the belief that there is no longer any risk of transmission based on the “U = U” medical standard and the use of protective measures. Furthermore, the societal stigma associated with AIDS has a significant impact on the willingness of PLHIV to disclose. This current “AIDS stigma” often deters infected individuals from actively fulfilling their disclosure obligations. Therefore, it is essential for all stakeholders in public health to strengthen education and awareness, enhance societal inclusivity, and reduce the social exclusion of PLHIV. Based on the “U = U” standard, the legal basis for disclosure obligations should shift from disease prevention to the rights and duties within the legal relationships of the individuals involved. When determining the subjects of disclosure, a distinction should be made between spouses and sexual partners, as disclosure to spouses is a specific expression of marital rights. Lastly, non-disclosure should not be equated with intentional transmission, and PLHIV who fail to disclose their status should not face additional legal responsibilities apart from the intervention of indirect disclosure. However, actions of intentional transmission should be severely punished through criminal measures such as charges of intentional harm, among others.

## Data Availability

The data that support the findings of this study are not openly available due to reasons of sensitivity and are available from the corresponding author upon reasonable request.

## Footnotes

1 Article 10 of the Marriage Law of the People’s Republic of China: A marriage is invalid if one of the following circumstances occurs: (2) suffering from a medical condition before marriage that is considered unfit for marriage and remains uncured after marriage.

2 Article 1053 of the Civil Code of the People’s Republic of China states: If one party suffers from a serious illness, they shall truthfully inform the other party before registering the marriage; if they fail to do so, the other party may request the People’s Court to annul the marriage. The request for annulment of marriage shall be made within one year from the date when the other party becomes aware or should become aware of the grounds for annulment.

3 Article 9 of the Law of the People’s Republic of China on Maternal and Infant Health: Upon pre-marital medical examination, if either party is found to be suffering from a designated infectious disease during the infectious period or a relevant mental illness during the onset period, the physician shall provide a medical opinion; both the man and woman intending to marry shall postpone the marriage. Article 38 of this Law defines the following terms: Specified infectious diseases refer to HIV/AIDS, gonorrhea, syphilis, leprosy, and other infectious diseases specified in the Law of the People’s Republic of China on the Prevention and Control of Infectious Diseases, as well as other infectious diseases recognized by medical science as affecting marriage and reproduction.

4 Article 38 of the “Regulations on the Prevention and Control of HIV/AIDS” in China states: (2) PLHIV shall promptly inform individuals with whom they have sexual relations of their infection or illness.

5 Article 1053 of the Civil Code of the People’s Republic of China states: If one party suffers from a serious illness, they shall truthfully inform the other party before registering the marriage; if they fail to do so, the other party may request the People’s Court to annul the marriage. The request for annulment of marriage shall be made within one year from the date when the other party becomes aware or should become aware of the grounds for annulment.

6 The alliance, initiated by Mr. Bai Hua, a well-known advocate for HIV prevention, organizes PLHIV in various regions and industries through online groups, forming a nationwide network social organization.

7 Article 1053 of 2021 Civil Code: If one of the parties suffers from a serious disease, he shall truthfully inform the other party of such disease prior to marriage registration; where such information is not truthfully provided, the other party may apply to the people’s court to annul the marriage.

8 Hangzhou Xiaoshan District People’s Court of Zhejiang Province (2014) Hangxiao Civil First Instance No. 828.

9 Article 38 of the “Regulations on the Prevention and Control of HIV/AIDS” in China states: (2) PLHIV shall promptly inform individuals with whom they have sexual relations of their infection or illness.

10 Yongzhou Lengshuitan District People’s Court of Hunan Province (2015) Yongleng Civil First Instance No. 2896.

11 Wuhou District People’s Court of Chengdu City, Sichuan Province (2014) Wuhou District First Instance No.1314.

12 Article 1041 of 2021 Civil Code: The family and marriage are under the protection of the state. The marriage system based on the principles of marital freedom, monogamy, and equality between men and women shall be implemented; Article 1042 of 2021 Civil Code: Prohibiting arranged marriages, the buying and selling of marriages, and any other actions that interfere with the freedom of marriage.

13 Article 38 of the “Regulations on the Prevention and Control of HIV/AIDS” in China states: “(2) PLHIV shall promptly inform individuals with whom they have sexual relations of their infection or illness. (3) When seeking medical treatment, they shall truthfully inform the attending physician of their infection or illness.”

14 In the “Regulations on the Prevention and Control of HIV/AIDS in Guangxi Zhuang Autonomous Region,” Article 28 stipulates, “People living with HIV (PLHIV) and AIDS patients shall fulfill the following obligations: (5) promptly inform their spouses or individuals with whom they have sexual relations of their infection or illness.” In the “Regulations on the Prevention and Control of HIV/AIDS in Yunnan Province,” Article 20 specifies, “Infected individuals and patients shall promptly inform their spouses or sexual partners of their HIV infection. In cases where the individual fails to provide this information, medical and healthcare institutions have the right to do so.”

15 Article 1041 of 2021 Civil Code: The family and marriage are under the protection of the state. The marriage system based on the principles of marital freedom, monogamy, and equality between men and women shall be implemented; Article 1042 of 2021 Civil Code: Prohibiting arranged marriages, the buying and selling of marriages, and any other actions that interfere with the freedom of marriage.

16 Article 38 of the “Regulations on the Prevention and Control of HIV/AIDS” in China states: (2) PLHIV shall promptly inform individuals with whom they have sexual relations of their infection or illness.

17 Article 1053 of 2021 Civil Code: If one of the parties suffers from a serious disease, he shall truthfully inform the other party of such disease prior to marriage registration; where such information is not truthfully provided, the other party may apply to the people’s court to annul the marriage.

18 In the “Regulations on the Prevention and Control of HIV/AIDS in Yunnan Province,” Article 20 specifies, “PLHIV/AIDS shall promptly inform their spouses or sexual partners of their HIV infection.”

19 Article 38 of the “Regulations on the Prevention and Control of HIV/AIDS” in China states: (2) PLHIV shall promptly inform individuals with whom they have sexual relations of their infection or illness.

20 Article 39 of the “Regulations on the Prevention and Control of HIV/AIDS” stipulates: “Without the consent of the individual or their legal guardian, no entity or individual shall disclose the names, addresses, workplaces, portraits, medical history, or any other information that may reveal their specific identity of people living with HIV (PLHIV), AIDS patients, and their family members.”

21 Article 1053 of 2021 Civil Code: If one of the parties suffers from a serious disease, he shall truthfully inform the other party of such disease prior to marriage registration; where such information is not truthfully provided, the other party may apply to the people’s court to annul the marriage.

22 Article 1043 of 2021 Civil Code: …spouses shall be truthful to each other, respect each other, and love each other mutually…

23 Article 1042 of 2021 Civil Code: …prohibits married individuals from cohabiting with someone other than their spouse.
